# Health Tract, No. 100

**Published:** 1865

**Authors:** 


### HEALTH TRACT, No. 100.

### SOLDIERS CARED FOR.

Out of one thousand soldiers, one hundred and four are sick; this is the constant proportion, as reported by the Sanitary Commission. The autumn always increases the number, by reason of the hot days and cool nights, causing diarrheas and dysenteries, of every shade and degree. One yard and a half of stout woolen flannel, fourteen inches broad, worn, from August to November, tightly and constantly around the abdomen, in such a way that it will be double in front, with bits of tape strongly sewed on one end, and about one yard from the other, according to the size of the person, for convenience of tying, would do more toward preventing bowel-complaints among our brave and self-denying soldiers, than all known human means besides. This simple device arrested the onset of cholera, in three days, in one of the largest divisions of the Prussian army, when the terrible scourge last visited Europe. Let every family who has a member in the army, forward such an article on the instant of reading this; if you can do no better, send an old worn petticoat, for, by reason of its softness and pliability, it is better than any thing else. Let every mother who reads this, and who may have no son or other relative bravely battling for the perpetuity of our glorious Union, send one abdominal bandage, to be given to some worthy soldier who has no mother, no sister, no wife, to exercise these kindly cares for him. And let the generous rich, of whom there are so many among us—the Astors, the Aspinwalls, the Minturns, the Stuart Brothers, and those like them—be assured that it is impossible to spend an equal amount of money as efficiently, in any other way. One man who has been in the army twelve months is worth *now* two raw recruits; hence one dollar's worth of good woolen flannel for one of them, or even an old petticoat, by keeping such soldier healthy in the field, will be worth more than the fifty dollars bounty paid for the two recruits, under the present exigencies of the case.

Winter is coming; let the sisters and mothers of the soldiers begin to knit two or three pairs of thick woolen socks, to be forwarded to each son and brother by the first of October; let the toes and heels be double knitted, or sheathed with the blue cloth of some worn-out coat or pantaloons, cautioning the soldier to keep the toe-nails closely trimmed, so as to prevent the cutting of the socks.

Begin *at once*, and put up in quart tin cans, to be forwarded *at intervals*, (for if sent in large quantities at a time, they will be wasted or too lavishly used,) pickled cucumbers and cabbage. Onions are represented by physiologists to be among the most wholesome and nutritious of all the vegetable products, besides their immediately invigorating and enlivening effects. If a gallon of onions could be sent to each soldier, once a month, in addition to a quart of pickled cucumbers or cabbage, scurvy, already beginning to manifest itself, would be unknown. And if it could be felt how grateful a quart tin can of preserved berries, tomatoes, or fruits, would be to a soldier who does not see such things, preserved or fresh, sometimes for months together, their sisters, and mothers, and cousins, and wives would spare no little pains to prepare a good supply for months to come, and would begin to send them on the instant.

